# Artificial Rearing of Atlantic Salmon Juveniles for Supportive Breeding Programs Induces Long-Term Effects on Gut Microbiota after Stocking

**DOI:** 10.3390/microorganisms9091932

**Published:** 2021-09-11

**Authors:** Camille Lavoie, Kyle Wellband, Alysse Perreault, Louis Bernatchez, Nicolas Derome

**Affiliations:** 1 Department of Biology, Laval University, Québec, QC G1V 0A6, Canada; lavoie.c22@gmail.com (C.L.); alysse.perreault@gmail.com (A.P.); louis.bernatchez@bio.ulaval.ca (L.B.); 2 Institut de Biologie Intégrative et des Systèmes (IBIS), Laval University, Québec, QC G1V 0A6, Canada; 3 Department of Biology, Canadian Rivers Institute, University of New Brunswick, Fredericton, NB E3B 5A3, Canada; kyle.wellband@gmail.com

**Keywords:** 16S rRNA subunit, Atlantic salmon, artificial rearing, microbial ecology, microbiota, supportive breeding

## Abstract

In supportive breeding programs for wild salmon populations, stocked parr experience higher mortality rates than wild ones. Among other aspects of phenotype, the gut microbiota of artificially raised parr differs from that of wild parr before stocking. Early steps of microbiota ontogeny are tightly dependent upon environmental conditions, both of which exert long-term effects on host physiology. Therefore, our objective was to assess to what extent the resilience capacity of the microbiota of stocked salmon may prevent taxonomic convergence with that of their wild congeners after two months in the same natural environment. Using the 16S SSU rRNA marker gene, we tested the general hypothesis that environmental conditions during the very first steps of microbiota ontogeny imprint a permanent effect on later stages of microbiota recruitment. Our results first showed that gut microbiota composition of stocked and wild parr from the same genetic population, and sharing the same environment, was dependent on the early rearing environment. In contrast, skin microbiota in stocked individuals converged to that of wild individuals. Taxonomic composition and co-occurrence network analyses suggest an impairment of wild bacteria recruitment and a higher instability for the gut microbiota of stocked parr. This study is the first to demonstrate the long-term effect of early microbiota ontogeny in artificial rearing for natural population conservation programs, raising the need to implement microbial ecology.

## 1. Introduction

It is now well established that host-associated microbes play an essential role for the development of immune, digestive, and metabolic functions in a substantial proportion of, if not every living, organism on Earth. In particular, early life associated bacteria influence host physiology and gene expression through the epigenetic modification patterns and the first colonizing bacteria would have a tremendous long-term contribution to the metabolism of the host [1,2,3,4]. The first bacteria and other microorganisms that colonize every surface of the body are either transmitted vertically, by the mother [5,6,7,8,9], or horizontally, by the environment [5,10,11,12,13]. The latter is especially true for aquatic species hatching from eggs, since they are surrounded by microbial communities in the water. The microbiota ontogeny is influenced by biotic (host genotype [14,15,16,17,18], life stage cycle [19,20], population density [13,21] and diet [9,22,23,24,25,26]) as well as abiotic factors (water chemistry, temperature, and xenobiotics including antibiotics [13,21,27,28,29,30,31]). 

Given that pioneering microbiota are largely involved in controlling host gene expression during the early life stages, it would be expected that exposure to different abiotic factors associated with rearing conditions would translate into distinct host–microbiota interaction patterns, which in turn drive divergent physiological host phenotypes [22,32]. Therefore, microbial ecology studies are relevant in the context of supportive breeding programs, where eggs issued from wild breeders are incubated in captivity until reaching juvenile stage, in order to be released into the natural environment. Such stocking strategy is commonly performed in species such as Atlantic salmon (*Salmo salar*). For example, the demographic decline of *S. salar* populations in the Province of Quebec, Canada, led to the implementation of governmental restoration programs such as artificial supplementation (COSEWIC, 2010), which consists of incubating eggs and raising juveniles in a controlled environment and introducing them in nature, mostly at the parr life stage (i.e., juveniles that have spent at least one summer in hatcheries) [32]. In order to preserve the genetic integrity of locally adapted salmon populations [33], introduced juveniles are generated from wild breeders caught in the targeted population. Nevertheless, acclimation to hatchery conditions causes an overall reduced fitness of stocked fishes compared to wild ones [34,35,36,37]. We previously documented noticeable discrepancies in gut microbiota composition between wild and hatchery raised juveniles destined for stocking [32]. Captive juvenile microbiotas were enriched with lactic acid bacteria but lacking in key strains found in their wild counterparts. The extent of the alternative microbiota occurring in hatcheries suggests that the physiology of juveniles might be affected by their microbiota prior to their introduction into the river [32], which in turn may confer a disadvantage after their release into the wild environment.

However, it is still unclear whether the difference in microbiota of stocked fish persists and would affect the host physiology after stocking. Previous studies have reported a modification of the microbiota following the introduction of a new diet in fishes [22,38], but few studies have documented the fate of the microbiota when the host is exposed to a new environment. A study conducted on the microbiota during the smoltification process showed that microbial equilibrium is disrupted when the host undergoes a drastic change in environment [39]. In fish, the reshaping of the microbiota composition also occurred after a stress induced disruption, indicating that microbiota can adapt to a new environment by modifying its functional and taxonomic profile to its new requirements [40]. In addition, early bacterial communities, host physiology, and environmental filtering have been reported to affect the colonization process later in life [41,42,43]. This finding led to our hypothesis that stocked parr microbiota would be maladapted due to irreversible priority effects during early ontogeny in hatchery, preventing them from recruiting key wild bacteria after stocking into the river.

Here, we report the first study assessing the long-term effects of the early life associated environment on microbiota composition by comparing the microbiota of stocked and wild parr two months after stocking in the natural environment, where stocked parr were introduced as unfed fry (that is with their yolk sac still attached). Based on both neutral and deterministic processes that shape the sequential recruitment of bacterial community in fishes [10,41] and on the partial plasticity of the gut microbiome following changes in diet or environment [22,38,39], our results suggest that stocked parr microbiota will show different patterns in terms of composition and structure compared to wild parr, and changes would differ by body compartment [28,40,44]. The microbiota taxonomic composition was characterized in cutaneous mucus, intestinal tract (i.e., the gut) and intestinal content (i.e., the digesta) samples of wild and stocked parr. Three different intestinal segments (anterior, median, and posterior segments) were targeted separately [26,45]. Overall, the most salient taxonomical and functional differences between stocked and wild parr originating from the same genetic population were identified in gut microbiota. Specific tissue responses were also identified, with cutaneous mucus tissue the least resilient compartment (i.e., being the most similar between stocked and wild parr), as opposed to the adherent (intestinal tract) and transient (intestinal content) gut communities that were significantly different between captive and wild parr. Neither host genetic diversity nor stomach contents were found to be associated with microbiota differences between stocked and wild parr. 

## 2. Materials and Methods

### 2.1. Sample Collection and Preparation

Stocked parr originated from the artificial spawning of wild breeders that were captured in the Puyjalon River (Havre-Saint-Pierre, QC, Canada) and kept in captivity in the LAboratoire Régional en Sciences Aquatiques (LARSA) of Laval University (Quebec, QC, Canada) for a period ranging from 1 to 2 years before spawning. The spawning took place between October and November 2015. The progeny hatched between 21 March and 30 May 2016, and unfed fry (still with their yolk sac attached) were kept in incubators until stocking in the Puyjalon River of unfed fry, between 24 and 29 July. Between 23 September and 29 September 2016, 93 parr were sampled in the Puyjalon River (50° 28′ 11.299″ N, −63°28′4.19″ W to 50° 27′ 37.40″ N, 63° 28′ 45.6″ W), Quebec, Canada by electrofishing and dragnet fishing. Environmental bacterial community composition was characterized by sampling one liter of water collected using sterilized Nalgene bottles from Puyjalon River. Samples were collected in triplicate, one meter below the surface. Water was filtered with peristaltic filtration equipment (Cole Parmer, Thermofisher Scientific, Vernon Hills, IL, USA) through a combination of 3.0 µm (to exclude suspended material and larger eukaryotic cells) and 0.22 µm (to collect bacteria community) sterile nitrocellulose membranes. Collection of fish tissue samples occurred as follows: parr were euthanized using the physical method called “flick” [45], cutaneous mucus was collected with sterile cell scraper and stored in sterile Eppendorf microtube, caudal fins were clipped and conserved in 95% ethanol solution for genotyping, and fishes were flash frozen in the field using flaked dried ice and stored in a −80 °C freezer until dissection. For each sample, stomach contents, gut, and feces from anterior, median, and posterior segments of the intestinal tract were aseptically collected (Supplementary Materials, Figure S1). The partition of the gut and the feces allowed the separated analysis of the bacteria associated with the intestinal mucosa (i.e., bacteria adherent with the gut), and the transient gut content (i.e., bacteria in transition in the digesta). To ensure the separated analysis of the adherent and transient bacteria, each gut segment was rinsed with a 1× PBS sterile solution after collection of the intestinal content. 

To determine whether the parr originated from supplementation breeding or from the natural environment, a panel of 17 microsatellites loci (sequences and primers are presented in the Supplementary Materials, Table S8) [46,47,48,49,50] was amplified under three PCR reactions using the DNA extracted from the fins using the Aljanabi and Martinez protocol [51]. PCR amplified markers were migrated on a 3500 Genetic Analyzer sequencer (Applied Biosystems, Life Technologies Corporation, Beverly, MA, USA). The PCR conditions and the volume for each primer are presented in the Supplementary Materials (Genotyping protocole, Table S1). After the migration, the allelic composition was investigated using the GeneMapper v.4.1. software (Thermo Fisher, Waltham, MA, USA). Parental assignment was performed using the Cervus v.3.0.7 software (Field Genetics Ltd., London, UK) [52] to determine whether the parr originated from artificial spawning. Parents were assigned to parr at a confidence threshold of 95%. To validate the genetic background homogeneity between stocked and wild parr, genetic diversity was determined by calculating the allelic richness and observed heterozygosity using the adegenet v.2.1.1 and heirfstat v.0.04-22 R packages [53,54]. The analysis was performed by combining individuals sampled in 2016 and 2017 to obtain an optimal sample size of a minimum of 30 individuals per group, since it is not possible to adequately calculate the genetic diversity of a small sample size (11 wild and nine stocked parr for 2016). DNA extraction of stomach contents, cutaneous mucus, gut, digesta, and water samples for the microbiome characterization was undertaken by modifying the Aljanabi and Martinez protocol [51] in order to optimize the bacterial cell lysis. Additional steps for DNA extractions are presented in the Supplementary Materials (Genotyping protocole for microbiota). Then, a first PCR amplification (PCR1) was performed on the water, mucus, gut, and digesta samples using the 519-F and 745-R primers (Sigma-Aldrich, St. Louis, MO, USA), targeting the V4 region of the universal microbial marker 16S small subunit ribosomal RNA (16S SSU rRNA) gene (Supplementary Materials, Tables S2 and S3). A second PCR (PCR2) (i.e., barcoding step) was performed using a two marker combination to identify each sample. Diet characterization: the cytochrome c oxidase subunit I (COI) was amplified from stomach samples with the indexed jgHCOI [55] and mLCOI [56] primers (Sigma-Aldrich, Darmstadt, Germany). PCR products were purified using AMPure XP beads (Beckman Coulter, Brea, CA, USA). For both 16S SSU rRNA and COI amplifications, reagents and PCR settings are described in the Supplementary Materials (Genotyping protocole, Tables S1–S5). Paired-end amplicon libraries were sequenced on a MiSeq Illumina platform using a read length of 2 × 300 pb and V3 kit reagent at the Institut de Biologie Intégrative et des Systèmes (IBIS) of Laval University, Quebec, QC, Canada (http://www.ibis.ulaval.ca/en/home/).

### 2.2. Sequences and Statistical Analyses

Paired-end sequences of 16S SSU rRNA gene were processed using the divisive amplicon denoising algorithm (DADA2; [57]) package under R (v.3.4.1). Assembly of paired reads was conducted as follows: Forward reads were truncated at a length of 260 bp and reverse reads at 205 bp, ensuring a phred score ≥ 20. Maximum expected error (maxEE) parameters were set to 4 for both forward and reverse reads. Filtering and trimming steps conserved 71.46% of the total sequences, which were used for the subsequent steps. After the dereplication and learning error rates steps, paired reads were merged, from which a sequence table of amplicon sequence variants (ASVs) was obtained. Bimeras sequences were then removed, and the taxonomical assignation was performed using the SILVA v.132 reference database on the remaining merged sequences. After the taxonomic assignment, a phylogenetic tree was constructed using the phangorn R package to align filtered sequences and to create a tree using the neighbor joining method. 

The analysis of microbial community composition and structure was performed using the Phyloseq [58] package under R. The composition tells which bacteria are present in the microbiota whereas the structure gives information about the distribution and interaction of the bacteria. The latter is evaluated with the core microbiota, alpha diversity, and network interactions analysis. Because samples with a low ASV number (<100) are suspected to result from PCR errors, a quality filtering was undertaken by removing those samples, and mitochondria reads were removed in all samples. In this study, one sample from the median gut of wild parr (MM_283) and one sample from the water (EA_016) had <100 ASV. To compare the microbiota composition between groups, permutation-based multivariate analysis of variance (PERMANOVA) of the weighted and unweighted UniFrac distances were undertaken. UniFrac distances of digesta and gut microbiota were visualized with a principal coordinate analysis (PCoA). Bar plots of the 10 most abundant families from the water, cutaneous mucus, gut, and digesta of stocked and wild parr were constructed to visually compare the microbiota composition. A core microbiota defined as present in 70% of samples was calculated using the “compute_core_microbiome.py” under QIIME [59] for gut samples. To further the analysis of the core microbiota of gut samples, a multiple analysis of variance (ANOVA) was performed after testing the homoscedasticity of the variances (Bartlett test, *p*-value < 0.05) on the abundance of each genus that appeared in the core microbiota using the origin and the sample type as covariates. When significant, post-hoc Tukey honest significant difference (HSD) tests were performed to identify the factors that were actually different. The functional profile potentially conferred by the gut microbiota (i.e., the body compartment being the most correlated with host metabolism) was estimated by performing a functional inference analysis based on taxonomic profile using METAGENassist [60]. Briefly, functional inference analysis consists of the taxonomic-to-phenotypic mapping of a bacterial community based on the BacMap [61], PubMed [62], and GOLD [63] databases. The ASVs from gut samples were filtered to exclude unassigned ASVs and ASVs that were at zero counts for 90% of the samples. Normalization of the reads was performed so that each sample could be compared to each other. To do so, read counts were normalized based on the total sum, and taxa abundance normalization was performed based on Pareto scaling. Inferred metabolism profiles of the gut microbiota were visualized with a heat map generated using the Pearson distance.

The structure of the microbiota was also evaluated by determining the diversity (Shannon) and richness (Chao1) from the microbiota of every sample type (cutaneous mucus, digesta, gut, and water) and both parr’ origin (wild vs. stocked). To assess whether significant differences in Shannon, Chao1 indexes can be identified and explained by the origin of the fishes, an analysis of variance (ANOVA; on Shannon) or its non-parametric equivalent (Kruskal-Wallis; on Chao1) was performed. The statistical analysis included the sample type, origin of the fishes, and the interaction of both factors (sample type and origin). To determine which means were significantly different from each other, a Tukey HSD test (for Shannon index) and a post-hoc analysis for Kruskal–Wallis (using the PMCMR package for Chao1 index) were conducted. The contribution of water microbial community and exclusive taxa in the samples was evaluated with the online tool Venny 2.0 [64]. To do so, ASVs that had at least one read across all samples and an average abundance >0.01% were compared between water, wild, and stocked parr microbiota. The abundance occupied by exclusive taxa in the samples was then compared by performing an ANOVA to determine whether the abundance of exclusive taxa varied across wild and stocked parr. Finally, a correlation analysis of taxa that were at a higher abundance than 0.01% in stocked and wild parr’ intestinal tract (gut and digesta combined) microbiota was performed and visualized under Cytoscape (v. 3.5.1.) using the Spearman correlation coefficient with a threshold value of 0.5 and a Bonferroni correction > 0.05 for significant correlations.

## 3. Results

Parentage assignment allowed for the determination of parental couples with a 95% confidence for each parent. Among the 93 sampled parr, 11 were assigned to known captive breeders, confirming their origin from the supportive breeding program. In order to have a balanced design for further analysis, an equal number of wild parr was randomly selected for the characterization of their microbiota. The mean allelic richness calculated for wild parr was 5.16 (S.E. 1.30) and 5.14 (S.E. 1.23) for stocked ones, whereas observed heterozygosity was 0.79 (S.E. 0.12) for wild parr and 0.81 (S.E. 0.13) for stocked parr. The genetic diversity analysis showed no significant differences between the two groups of parr (T test, T = 0.10567 and *p*-value = 0.9172 for allelic richness; T = 0.7272 and *p*-value = 0.4776 for observed heterozygosity, Supplementary Figure S2).

**Figure 1:**
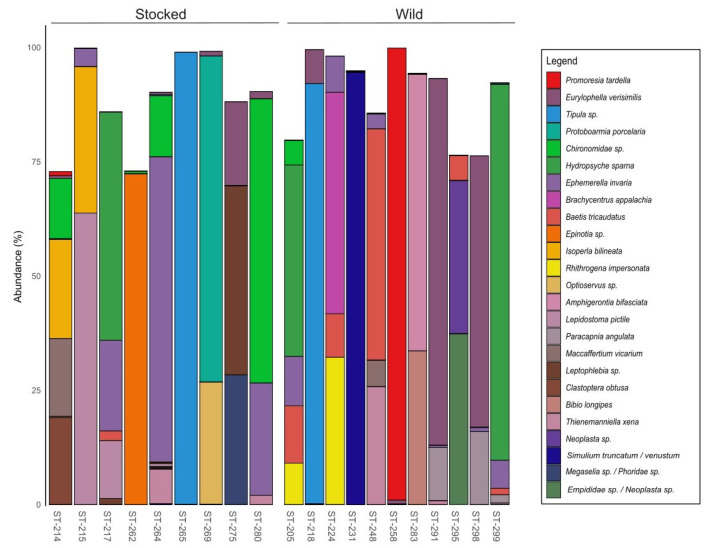
The 25 most abundant eukaryotic species identified by the amplification of the biomarker Cytochrome Oxydase I (COI) from the stocked and wild parrs’ stomach contents highlight the high interindividual variability between both groups.

Stomach contents COI metabarcoding: The sequencing of COI amplicons resulted in 575,534 reads per sample, which was reduced to 412,484 reads once the host contamination was removed. COI metabarcoding analysis performed on Bray Curtis distance matrices revealed no differences in prey profiles in captive and wild parr (PERMANOVA, F = 0.0578, *p*-value 0.276) as well as a high interindividual variability (Supplementary Figure S3). The stomach contents were mainly composed of various species of annelids and arthropods, the most abundant belonging to *Eurylophylla* (13.44%), *Hydropsyche* (11.28%), and *Promoresia* (8.99%) for wild parr and to *Ephemerella* (12.81%), *Tipula* (11.01%), and *Chironomidae* (9.91%) for stocked parr. The 25 most abundant preys can be visualized in Figure 1 and their respective frequency are described in the Supplementary Materials (Table S7).

Microbiota characterization with 16S SSU rRNA metabarcoding: After the taxonomical assignation and quality filtering, 8401 ASVs were distributed within 23 phyla and 715 bacterial genera. The PERMANOVA analysis performed on the weighted and unweighted UniFrac distances showed that the microbial community composition was influenced by the origin of the fishes, but was also dependent on the body compartments (Table 1). Overall, the origin had a significant effect on the proportion of the bacteria present in the microbiota of gut samples (weighted Unifrac; F = 5.8617, *p*-value = 0.003, unweighted Unifrac; F = 1.2358, *p*-value = 0.072) and on the taxonomical composition of the digesta microbiota (weighted Unifrac; F = 18822, *p*-value = 0.069, unweighted Unifrac; F = 1.8704, *p*-value = 0.002). The PCoA analysis was concordant with the PERMANOVA results, which revealed a clustering of samples for the weighted UniFrac distances of gut samples and unweighted UniFrac distances of digesta samples (Supplementary Figure S4). 

**Figure 2:**
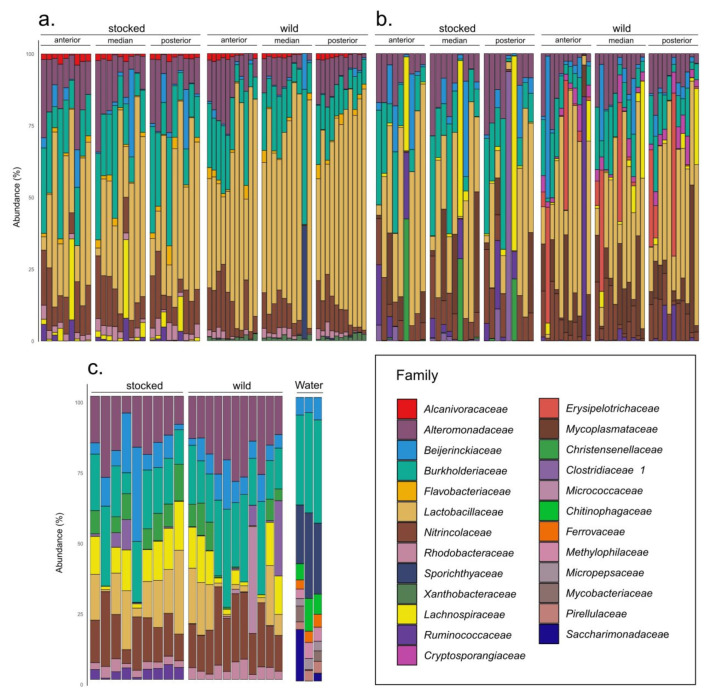
The normalized sum (%) of the 10 most abundant bacterial families from gut (**a**), digesta (**b**), mucus, and water (**c**). The bacterial composition for each sample type and origin suggests a different microbiome for parrs from different origin, especially for gut and digesta samples. Even though numerous families are shared between stocked and wild parr, bacterial abundance varies from gut samples. Digesta samples show distinct families that are not shared between captive and stocked parr such as *Cryptosporangiaceae* (wild only), *Erysipelotrichaceae* (wild only), and *Clostridiaceae*_1 (stocked only).

For each body compartment and parr origin, the relative mean abundance of the 10 most abundant families is indicated in Supplementary Materials (Table S6) and visualized in Figure 2. For cutaneous mucus, we identified wild parr exclusive taxa belonging to the *Chritensenellaceae* and *Micrococcaceae* families. Distinct families can also be visualized for gut samples (*Alcanivoraceae*, *Flavobacteriaceae*, *Sporichthyaceae*, and *Xanthobacteraceae* for one sample only) and digesta samples (*Gemmataceae*). In addition, wild parr digesta were characterized by the presence of *Erysipelotrichaceae*, *Mycoplasmataceae*, and *Pirellulaceae*. For stocked parr digesta, we found taxa from the *Isospheraceae* family that were not shared with the 10 most abundant families from the wild digesta. 

**Figure 3:**
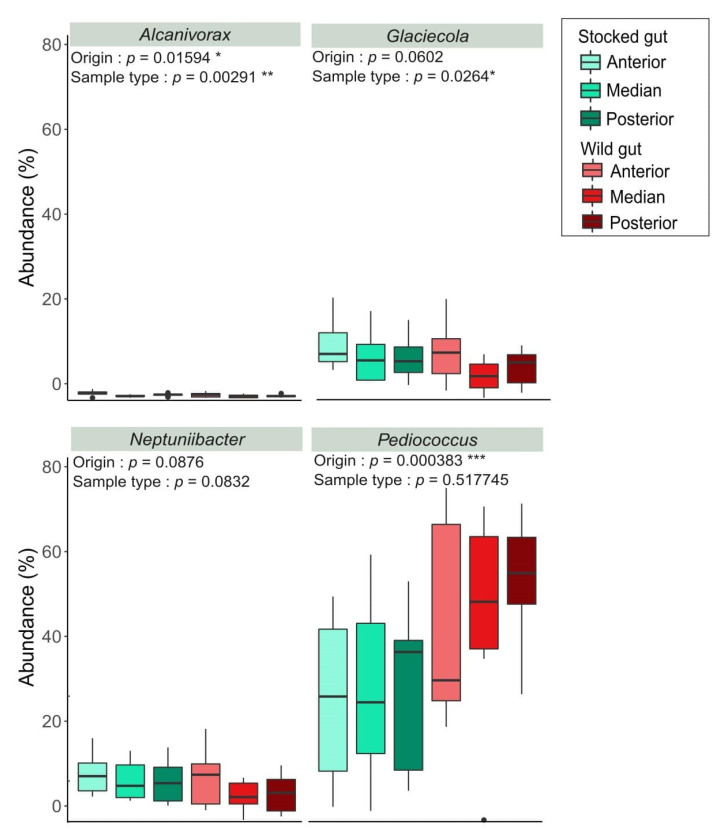
Abundance of the core microbiota taxa present in at least 70% of gut samples from parr originating from supportive breeding programs (stocked) or natural environment (wild). Multivariate analysis indicates either a significant (*Pediococcus* sp. and *Alvanivorax* sp.) or marginal (*Glaciecola* sp. and *Neptuniibacter* sp.) effect of the parr’s origin on taxa abundance. Sample type (anterior, median, or posterior compartment of the intestine) was identified to have a significant effect on taxa abundance for *Alcanivorax* and *Glaciecola* genera, but no interaction between the origin and the sample type was highlighted. “*” <0.05; “**”<0.01; “***”<0.001 “ ” n.s.

A core microbiota of taxa is present in at least 70% of gut samples identified bacteria from the genus *Pediococcus* (*Lactobacillaceae*), *Glaciecola* (*Alteromonadaceae*), *Neptuniibacter* (*Nitrincolaceae*), and *Alcanivorax* (*Alcanivoraceae*) (Figure 3). The multivariate analysis performed on the abundance of the core bacterial genus identified a significant contribution of the origin for the abundance of *Pediococcus* (F = 14.391, *p*-value = 0.000383) and *Alcanivorax* (F = 6.201, *p*-value = 0.01594), and a marginal effect on the abundance of the *Glaciecola* (F = 3.689, *p*-value = 0.0602) and *Neptuniibacter* (F = 3.029, *p*-value = 0.0876) genus. Although *Alcanivorax* and *Glaciecola*’s abundance were also affected by the sample type (F = 6.534, *p*-value = 0.00291 and F = 3.895, *p*-value = 0.0264, respectively), there was no interaction between the origin and the sample type. When compared with Tukey HSD test, the gut section (anterior, median, and posterior) did not show any differences for bacteria abundance, but was rather influenced by the origin or the sample type. Finally, core microbiota from digesta and mucus samples did not reveal any differentially relative abundant taxa between wild and stocked samples. 

**Figure 4:**
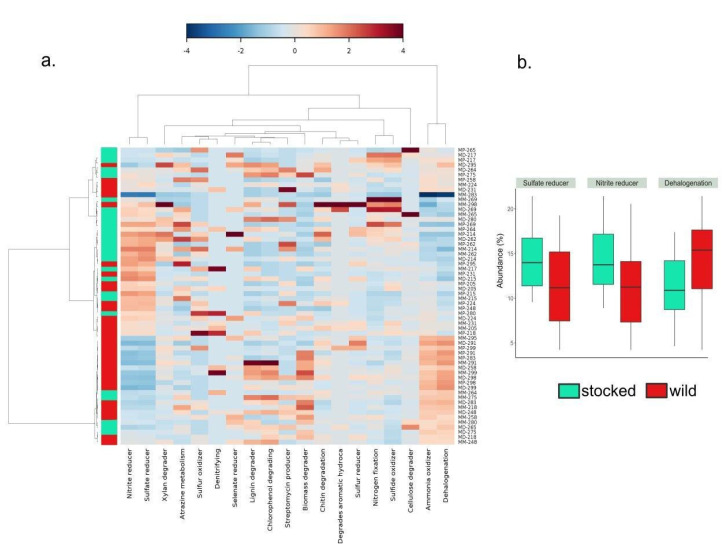
Functional inference performed with METAGENassist of gut samples shows a broad clustering of functions for stocked and wild parrs’ microbiota. (**a**) The heatmap, based on Pearson distances, highlights different functional profiles for different clusters regarding the parrs’ origin, where stocked (red) and wild (turquoise) parrs were separated in two groups according to their functional profile. The abundance of the inferred functions is proportional to the relative abundance of the taxa and are presented in the columns of the heat map (blue; low abundance, red; high abundance). Each line represents one sample from the gut (M; intestinal mucosa) for each compartment (P; proximal, M; median, D; distal). (**b**) Abundance (%) of inferred functions that are found at a significant different abundance for gut samples between captive and stocked parr (FDR < 0.05).

The functional inference performed with METAGENassist on gut samples resulted in a broad clustering of wild and stocked parr, where stocked and wild parr were more or less separated in two distinct groups according to their functional profile (Figure 4a). In addition, three functions were differentially represented between wild and stocked parr (t-test, FDR < 0.05), where the functions “sulfate reducer” and “nitrite reducer” were found to be at a significantly higher relative abundance for stocked fish (*p*-values = 0.00118 and 0.0012, respectively) and “dehalogenation” was significantly higher for wild parr (*p*-value = 0.0064) (Figure 4b). 

**Figure 5:**
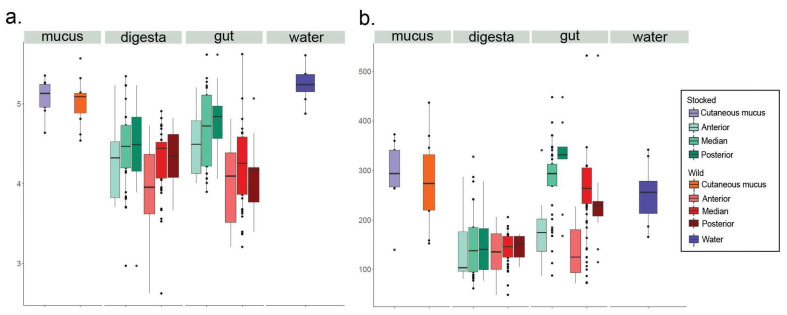
Alpha diversity calculated with Shannon (**a**) and Chao1 (**b**) indexes for every sample type regarding the parrs’ origin highlights a generally higher diversity for the stocked parrs’ microbiota, especially in gut samples. Chao1 was computed at the genus level.

Shannon index (Figure 5) was affected by both sample type (ANOVA, F = 6.987, *p*-value *=* 4.57 × 10^−10^) and origin (F = 12.924, *p*-value = 0.000161). No interaction between the sample type and origin was found (F = 1.336, *p*-value *=* 0.1845), suggesting that the effect of the origin was the same for every sample type. Overall, the Shannon index was higher within the stocked gut samples compared to the wild gut samples (Tukey HSD, *p*-value = 0.0013117), especially for the posterior segment (Tukey HSD, *p*-value *=* 0.03341), but was neither significantly different for both digesta and cutaneous mucus samples (Tukey HSD, *p*-value *=* 0.9651 and 1.0, respectively) nor for other gut sections. Finally, the Kruskall–Wallis analysis on Chao1 indicated no significant differences between stocked and wild parr when body compartments were compared.

**Figure 6:**
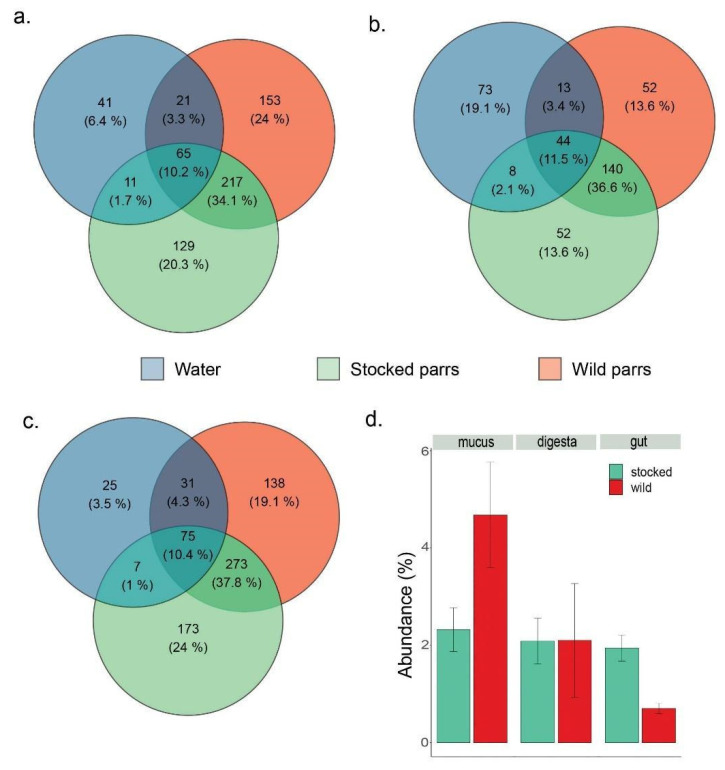
Venn diagrams analysis (**a**–**c**) performed with Venny 2.0 indicate the potential contribution to water microbiome in the parrs’ bacterial community. Diagrams represent the number of taxa over a mean abundance of 0.01% that are shared—or not—between water and each body compartment according to the parrs’ origin. Number of shared taxa from water and cutaneous mucus (**a**), digesta (**b**), or gut samples (**c**), and the proportion occupied by exclusive taxa (**d**) suggest an impairment in the recruitment of water microbiome in samples of stocked parrs, as the number of shared taxa with water is lower in gut and mucus for stocked samples than for the wild samples.

The analysis of shared and exclusive taxa revealed a contribution of the bacterioplankton as a source of bacterial strains to the cutaneous mucus, gut, and digesta microbiota (Figure 6). The portion of shared taxa with water was significantly higher for wild samples (3.4% for digesta, 4.3% for gut, and 3.3% for cutaneous mucus) than their stocked relatives (2.1% for digesta, 1% for gut, and 1.7% for cutaneous mucus, *p*-value = 0.010, Figure 6a–c). Exclusive taxa from gut samples showed a significantly higher abundance in stocked samples (Figure 6) (*p*-value ≤ 0.001). In contrast, the proportion of exclusive taxa from the mucus and digesta samples did not significantly differ between stocked and wild parr. 

**Figure 7:**
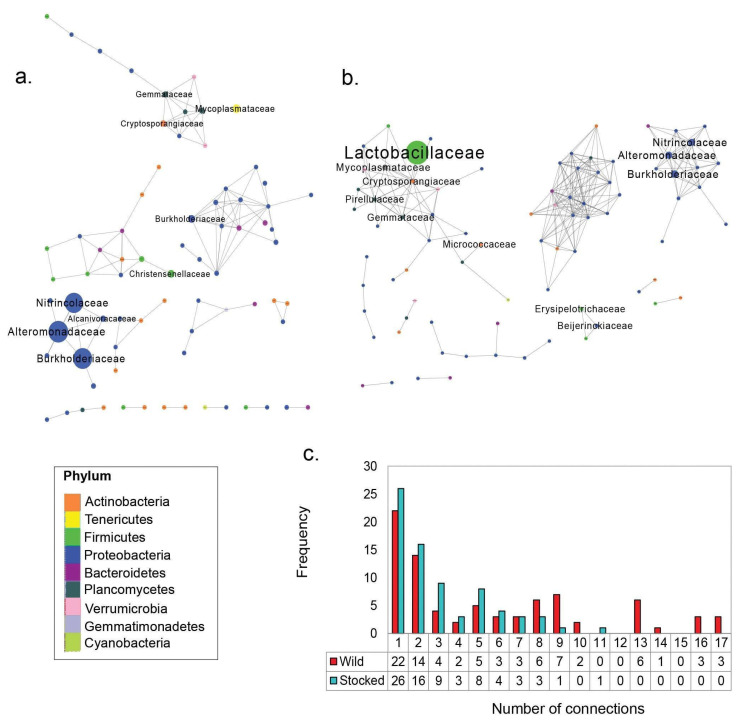
Co-occurrence network analysis from intestinal communities (gut and digesta). The co-occurrence was calculated based on Spearman correlations (>0.5, Bonferroni correction < 0.05) for taxa over 0.01% at the genus level. Twice as many significant correlations were found in the wild parr network (**b**) compared to stocked parrs (**a**). Main taxa with significant correlations are represented at the family level, where the size of the circles is associated with the relative abundance of the taxa within the microbiome and the color is associated to a Phylum. Co-occurrence network of wild parr microbiota suggests a higher stability, being characterized by a higher number of connections (**c**) and a higher number of interactions. The number of connections and their frequency, shown in Figure 7c, highlight the higher occurrence of nodes with more connections for the microbiota of wild parr.

The co-occurrence network analysis showed twice as many of significant correlations over a 0.05 threshold in wild parr than in their stocked relatives (228 vs. 113) (Figure 6). The interaction network of the wild parr’ gut microbiota was characterized by more nestedness for wild parr: higher clustering coefficient (0.476 vs. 0.383), network density (0.070 vs. 0.042), and the number of edges per node (bacteria phyla; Figure 7c).

## 4. Discussion

Physiological, metabolic, epigenetic, and microbiota alterations have been associated with hatchery rearing for supportive breeding and conservation programs [32,34,35,36,37,65,66,67,68,69,70,71]. However, it was not known to what extent microbial discrepancies between captive and wild fish observed prior to the stocking event would fade after their introduction into the wild environment. This question is particularly salient because the resilience of microbiota is variable according to the body site (e.g., skin versus gut [28,40]), and is modulated by factors such as environmental filtering [14,22,29,31,41], diet [10,24,25,26,27] as well as host physiology [17,32] and genotype [15,16,17,18,72]. The present work is of prime interest as it suggests that host physiology, and consequently host survival, could be associated with the remaining discrepancies of microbiota composition caused by artificial rearing of parr that originate from supportive breeding programs.

In our study, we demonstrated that strong deterministic processes that are neither related to the population’s genotype nor the diet (stocking of unfed larvae), occur at early life stage cycles. Specifically, hatchery rearing was associated with a diminished capacity of stocked parr to recruit wild key commensals such as *Pediococcus*. Hatchery rearing also induced an immature and unstable microbiota, as observed by the lower diversity of commensals in their gut and by the weak connectivity in the bacteria network. This statement is supported by our results, as stocked fish still have a different gut microbiota than their wild relatives after spending two months in the same river habitat. In addition, gut wall and digesta community analysis revealed the most salient differences in the taxonomic composition. In contrast, skin mucus microbiota of hatchery-raised juveniles was much less resilient to the change in environment as shown by taxonomic convergence with their wild counterparts.

The fact that the origin (hatchery vs. wild) best explains the differences observed in the microbiota is striking because; (i) stocked and wild individuals shared the same genetic background and showed similar patterns of genetic diversity; (ii) stocked individuals were not fed prior to their introduction into the river; and (iii) both groups had similar stomach contents at the time of sampling. The first argument rejects the host genotype effect, whereas the second and third ones discard the diet effect. 

It is not surprising that the gut microbiota of two month juveniles is still imprinted by its initial environment, although sharing the same environment as their wild relatives after spending two months in the wild, as fish gut bacterial colonization starts as soon as the larvae open its mouth [25]. Therefore, gut colonization started from the last two months spent in the hatchery. While stocked and wild parr’ microbiota shared bacterial species found naturally in Atlantic salmon populations [17,19,20,32], our results highlighted many differences in the microbial recruitment processes between stocked and wild parr, as the microbiota composition was significantly different, in addition to the abundance of the core microbiota taxa that were significantly or marginally different between both groups. It was previously suggested that stocking of unfed fry might help mitigate the acclimation occurring in hatcheries [36]. However, considering that ecological processes of microbial ecological drift and host physiology are key drivers of bacterial colonization processes [72] and that physiology and microbiota composition are modified by artificial rearing [32,34,35,36,37], our results support the hypothesis that early environmental conditions exert an influence on the gut microbiota ontogeny after stocking. This phenomenon was also reported in newborn chicken [43], where the recruitment capacity of allochthonous bacteria was altered by the pioneering microbiota. 

The microbiota discrepancy observed between wild and stocked parr could also translate into functional divergence, as suggested by the functional inference results. Thus, while it was not possible to assess the exact contribution of the core microbiome taxa to the different metabolic functions identified with METAGENassist, our results suggest that even after having spent two months in the same environment as wild parr, hatchery reared parr microbiota may not confer the same metabolic function to their host. Because microbiota interacts with host physiology both in terms of immune response and metabolic performance [1,2,3,4], our results support the relevance of monitoring microbial communities in hatcheries for supplementation programs.

In addition, the alpha diversity analysis revealed that the stocked parr’ microbiota still exhibited signs of immaturity comparatively to their wild counterparts. For instance, stocked parr microbiota is characterized with a higher diversity than that of their wild relatives. Once again, the highest discrepancy between stocked and wild parr was observed for the gut microbiota. Shannon diversity differences were explained by the origin (stocked vs. wild) and to some extent by the gut section, being much higher in the posterior section. The lower diversity observed in wild parr’ gut is concordant with the analyses on the core microbiota as it highlights a more homogenous microbiota, over-dominated by one bacterial species. In zebrafish (*Danio rerio*), early stages were characterized with the highest diversity relatively to the later stages [73]. As such, the lower diversity and the overdominance of *Lactobacillaceae* recorded in the gut microbiota of wild parr reveals a more mature microbiota, as opposed to the more diverse and less structured microbiota found in stocked parr guts. A similar contrast was observed in our previous study comparing captive and wild parr gut microbiota issued from the same population [32].

Colonization resistance to environmental bacteria was also observed in stocked parr by measuring the potential contribution of the bacterioplankton as a source to the host’s microbiota. This was also evaluated by observing the number of shared taxa with water and the different body compartments. Systematic lower number of environmental taxa shared with stocked parr microbiome was observed, regardless of the body compartment. It can therefore be hypothesized that the contribution of wild bacterioplankton to the microbiota ontogeny may be altered by deterministic processes that occurred in hatchery. In addition, the higher abundance of exclusive taxa in stocked parr gut microbiota could be indicators of earlier deterministic processes controlling bacterial recruitment during their incubation in hatchery. Taken together, our results also suggest that early microbiota ontogeny in hatchery translated into a partial colonization resistance to wild bacteria. 

The results of this study also highlight the contribution of bacterioplankton (indicated by common taxa in water and both wild and stocked parr samples) in the microbiota. The recurrent presence of taxa from the families *Beijerinckiaceae*, *Burkholderiaceae*, and *Sporichtyaceae* in skin mucus, gut, and digesta samples and their high abundance in water (belonging to the 25 most abundant families in the bacterioplankton, Figure 2) can be used as indicators of the contribution of bacterioplankton in the composition of microbiota. 

Finally, the co-occurrence network analysis suggests that wild parr’ gut microbiota is more stable and more resilient than that of their stocked relatives. To this respect, it has been proposed that co-occurrence network analysis can be used as an indicator of microbial community maturity and stability [74]: mature and stable communities are characterized by a high connectivity. Thus, a higher connectivity can be associated with a higher resilience capacity because more taxa can secure the same ecological functions. In contrast, less connected communities can be easily disturbed, as the suppression of one taxon translates into a transient state where no other taxa can rescue the same ecological function than the missing one [74]. Thus, our results support the hypothesis that hatchery rearing conditions, potentially those associated with decontamination and sanitary management, induce a long-term assemblage of a less resilient microbiota. 

To conclude, we hypothesized that the detected tissue specific response to environmental change between stocked and wild fish most likely reflect a difference in terms of resilience between compartments. For instance, bacterial communities from the cutaneous mucus are directly in contact with the environment. This promiscuity with the environment can result in a diminished resilience for cutaneous communities that are most likely driven by the environment rather than the host physiology itself, as observed in Sylvain et al. [10,14]. According to our results, the isolated microbial communities within the intestinal tract would rather be driven by deterministic processes, as described by the gut island theory [41], which can explain the higher divergence between stocked and wild parr. Furthermore, the incapacity of stocked parr to recruit key gut symbionts two months after their introduction into the wild underlines the incremental nature of early microbiota ontogeny, and the irreversibility of some priority effects at later stages [41,42]. 

Altogether, differences detected between wild and stocked parr microbiota suggest that early conditions to which parr are exposed during rearing continue to influence microbiota ontogeny for at least two months after stocking. Considering that stocked parr are subject to a significant physiological stress during the stocking process, the weak network connectivity observed in stocked parr two months after their introduction in the wild suggests that an immature and low resilience microbiota could compromise the parr acclimation to wild environment, at least during the very first months. 

## 5. Limitations

The results of the present study may be hampered by several experimental limitations. First, due to the numerous challenges of sampling parrs in a large river such as the Puyjalon River by electrofishing and dragnet fishing, quota limits for an endangered species, and most importantly to low survival of stocked individuals, it was not possible to sample more than 11 stocked individuals out of 93 parrs. However, the differences detected between the two groups were highly significant for gut and digestive content, so the hatchery effect was conservatively assessed. Given this moderate sampling size for stocked individuals, genetic diversity of the two groups was conducted over multi-year sampling (2016–2017) to achieve an optimal sample size of 30 individuals per group. The results unambiguously showed that there were no significant differences between the two groups, neither for allelic richness nor for observed heterozygosity. Finally, additional sampling is needed to measure to what extent microbiota composition differences between groups are still detectable at later life cycles. 

## 6. Conclusions

By assessing the long-term effects of the early life-associated environment on microbiota composition by comparing the microbiota of stocked and wild parr two months after the former’s introduction into the natural environment, our study highlights, for the first time, that stocked parr gut microbiota is still imprinted by hatchery environment, despite fish having been introduced prior to first feeding into the targeted river.

As numerous studies identified a role of the early colonizers in metabolic imprinting, microbial recruitment and the development of host immune and digestive system [1,2,3,22], our study stresses that microbial ecology in artificial rearing for supportive programs needs to be further investigated. For instance, the management of the water microbiome supported by the ecological theory, as proposed by De Schryver and Vadstein [75], is a promising sustainable development of the hatchery rearing method. Overall, a better understanding of the implication of bacterial communities in the low survival rate of stocked fishes needs further investigation on how the microbiota interacts with physiological and metabolic processes. 

## Figures and Tables

**Table 1:** PERMANOVA performed on the weighted and unweighted UniFrac distances indicates that the microbial community is influenced by the origin of the fish (stocked vs. wild), but are also dependent on the body compartments. Based on the taxa names and on their respective values, bacterial communities were significantly different between stocked and wild parrs, indicating that rearing methods have long-term effects on microbiota composition for fishes from the same genetic background.

	Weighted		Unweighted	
	F	*p*-Value	F	*p*-Value
Total microbiota	3.0842	0.011 *	1.6813	0.015 *
Digesta	1.8822	0.069	1.8704	0.002 **
Gut	5.8617	0.003 **	1.2358	0.072
Skin mucus	1.6048	0.133	1.0326	0.313

“*” <0.05; “**”<0.01; “ ” n.s.

## Data Availability

Data for this study are available at: 10.5061/dryad.6djh9w10c.

## Supplementary Materials

The Supplementary Material for this article can be found online at https://www.mdpi.com/article/10.3390/microorganisms9091932/s1, Figure S1: Body compartments and gut segments that were separated for the microbiota analysis of the intestinal tract, Figure S2: Genetic diversity for stocked and wild parrs calculated with individuals from year 2016 and 2017, Figure S3: The boxplot represents the distance to the centroid of the Bray Curtis distances within each group, based on an analysis of the multivariate homogeneity of dispersion (variances) of samples, Tables S1–S5: Genotyping protocole, Table S6: Relative abundance of the 10 most abundant families (%) from each body compartments in wild and stocked parrs. S.e.m. Standard error of the mean, Table S7: Mean relative abundance (%) of the 25 most abundant prey found in the stomach content of stocked and wild parrs. S.e.m. Standard error of the mean, Table S8: Primer sequence for genetic assignation.
