# Supplementary material for: Artificial Rearing of Atlantic Salmon Juveniles for Supportive Breeding Programs Induces Long-Term Effects on Gut Microbiota after Stocking
Source: Microorganisms. 2021 Sep 11;9(9):1932. doi: 10.3390/microorganisms9091932 (PMC8465833; doi:10.3390/microorganisms9091932)
Supplement: Supplementary file 1 [file microorganisms-09-01932-s001.zip › microorganisms-1357948-supplementary.pdf]

Supplementary materials

1. Genotyping protocol

a. Modifications for DNA extraction protocol from Aljanabi and Martinez 1997:

After the overnight incubation, 20 µL of Proteinase K was added and incubated for at least 2 hours at 50 °C before adding 4 µL of Rnase A (10 mg/L) for an incubation of one hour at room temperature. After adding the Isopropanol, the samples were incubated at -20 °C between one to 12 hours. The pellets were rinsed twice with cold 70 % ethanol, dried at 60 °C for 1 to 2 hours or at 37 °C overnight and resuspended in 100 µL sterile H_2O.

b. Microsatellites loci amplification protocol (Multiplex)

The Multiplex PCR kit (Qiagen, Product number 206145) were used to amplify the 17 chosen loci under three PCR reactions. For each PCR reactions, a volume of primers was added in order to obtain a final volume of 10 µL for the reaction. Primers quantity and PCR conditions for each reaction are described below and PCR conditions were the following: 95°C for 15 min (95°C for 30 sec; 58°C for 3 min; 72°C for 1 min)$_{35}$ 72°C for 30 min.

Tab. S1. Primers (reverse and forward) used for the microsatellites amplification protocol (total volume of 10 µL) and their respective quantity in µL for one reaction.

Reagent	Volume (µL)
PCR Multiplex 1	
Multiplex PCR kit	5
Ssa171 primer	0.2
Ssa197 primer	0.05
Ssa202 primer	0.3
Ssa1417 primer	0.15
SsaD85 primer	0.4
SsaD71 primer	0.4
DNA	2
PCR Multiplex 2	
Multiplex PCR kit	5
SsaD144 primer	0.6
Sssp1605 primer	0.5
Sssp2210 primer	0.1
Sssp2215 primer	0.25
Sssp2216 primer	0.05
DNA	2
PCR Multiplex 3	
Multiplex PCR kit	5
Sssp2201 primer	0.9
SsspG7 primer	0.1
MST-3 primer	0.25
SsaD58 primer	0.8
Ssa85 primer	0.03
SsaD486 primer	0.025
DNA	2

c. DNA migrations

For each sample, 1 µL of the Multiplex PCR product was diluted in 10 µL H_2O. Then, 2 µL of the dilution was mixed with 10 µL of a mix of 10 µL Formamide HiDi and 0.13 of GeneScan 500 LIZ (for Multiplex PCR 3, the product was GeneScan 500 ROX).

Modification for DNA extraction protocol from Aljanabi and Martinez for microbiota:

The protocol included a one-hour incubation at 37°C with 20 µL lysozyme (25 mg/mL) prior to the overnight incubation. In order to minimize the contamination with RNA, an additional incubation step with 4 µL Rnase A (10 mg/mL) for one hour at 37°C was performed at the end of the incubation process. A 300 µL volume of 6M NaCl solution was added to each sample and samples were vortexed and centrifuged at 4°C, 16 000g for 20 minutes. Supernatant were transferred, and another centrifugation was performed. An equal volume of cold isopropanol (stored at -20 °C) was added to the supernatant, mixed by inversion, and tubes were incubated for at least 30 minutes at -20 °C before being centrifuged for 20 minutes at 4°C, 16 000g. The pellet was washed with cold 70% ethanol (stored at -20 °C), dried and resuspended in 50-200 µL sterile milli-Q water. DNA concentration and quality were estimated by spectrophotometry (NanoDrop 2000, ThermoFisher Scientific).

Tab. S2. Used reagents (New England BioLabs) for PCR1 and PCR2 (total volume 50 µL) and their respective quantity in µL for processing one sample. Primers sequences: 519-F 5'-ACA CTC TTT CCC TAC ACG ACG CTC TTC CGA TCT CAG CMG CCG CGG TAA -3', 745-R 5'- GTG ACT GGA GTT CAG ACG TGT GCT CTT CCG ATC TGA CTA CHV GGG TAT CTA ATCC -3'.

Reagent	Quantity (µL)	
	PCR 1	PCR2
Q5 Buffer	10	10
dNTPs	1	1
519-F (10 µM)	2.5	-
745-R (10 µM)	2.5	-
Forward Primer, predetermined (10 µM)	-	1
Reverse Primer (markers) (10 µM)	-	1
«High GC enhancer»	10	10
DNA	2	2
Q5 Polymerase (New England Biolabs)	1	1
Sterile water	21	24

Tab. S3. DNA amplification conditions for PCR1 and PCR2. The « Denaturation », « Hybridation » and « Elongation » steps were performed 35 times for PCR1 and 12 times for PCR2.

Step	Temperature (°C)	Duration
Initial Denaturation	98	2 min.
Denaturation	98	10 sec.
Hybridation	60	30 sec.
Elongation	72	30 sec.
Final Elongation	72	10 min.

Tab. S4. Used reagents (Qiagen) for COI amplification (total volume 31µL) and their respective quantity in µL for processing one sample. Primers sequences (with **Illumina Nextera Adaptors**) : mLCOI 5'- **GTC TCG TGG GCT CGG AGA TGT GTA TAA GAG ACA G** TAI ACY TCI GGR TGI CCR AAR AAY CA -3', jgHCOI 5'- **TCG TCG GCA GCG TCA GAT GTG TAT AAG AGA CAG** GGW ACW GGW TGA ACW GTW TAY CCY CC -3'.

**Reagent**	**Quantity (µL)**
Master Mix	12
DNAse-free sterile H2O	12
jgHCOI (10 µM)	2
mLCOI (10 µM)	2
DNA	3

Tab. S5. DNA amplification conditions for COI. The « Denaturation », « Hybridation » and « Elongation » steps were performed 36 times.

**Step**	**Temperature (°C)**	**Duration**
Initial Denaturation	95	15 min.
Denaturation	94	30 sec.
Hybridation	52	1 min. 30 sec.
Elongation	72	1 min.
Final Elongation	72	10 min.

Tab. S6. Relative abundance of the 10 most abundant families (%) from each body compartments in wild and stocked parrs. S.e.m. Standard error of the mean.

Body compartment	Wild			Stocked		
	Family	mean (%)	s.e.m	Family	mean (%)	s.e.m
Gut	*Lactobacillaceae*	51	3.5	*Lactobacillaceae*	32	3.6
	Burkholderiaceae	13	1.1	*Burkholderiaceae*	16	1.5
	Alteromonadaceae	8.7	1.0	*Alteromonadaceae*	12	1.2
	Nitrincolaceae	8.0	1.0	*Nitrincolaceae*	10	0.9
	Rhodobacteraceae	1.4	0.2	*Beijerinckiaceae*	3.2	1.2
	Flavobacteriaceae	1.1	0.2	*Lachnospiraceae*	2.9	1.2
	Xanthobacteraceae	1.1	0.1	*Rhodobacteraceae*	2.4	0.2
	Alcanivoracaceae	0.9	0.1	*Alcanivoracaceae*	1.4	0.2
	Sporichthyaceae	0.7	0.6	*Flavobacteriaceae*	1.2	0.2
	Beijerinckiaceae	0.7	0.2	*Ruminococcaceae*	1.1	0.4
Digesta	*Lactobacillaceae*	27	3.3	*Lactobacillaceae*	24	3.9
	Mycoplasmataceae	8.4	1.8	*Burkholderiaceae*	11	1.7
	Burkholderiaceae	8.2	1.0	*Alteromonadaceae*	8.5	1.2
	Erysipelotrichaceae	7.6	2.6	*Nitrincolaceae*	7.4	1.0
	Alteromonadaceae	6.9	1.0	*Lachnospiraceae*	5.3	2.8
	Nitrincolaceae	6.5	0.9	*Mycoplasmataceae1*	4.6	1.6
	Beijerinckiaceae	3.6	1.5	*Beijerinckiaceae*	4.1	1.3
	Ruminococcaceae	3.4	2.5	*Clostridiaceae_1*	4.0	3.2
	Cryptosporangiaceae	2.6	0.3	*Ruminococcaceae*	3.2	1.1
	Lachnospiraceae	2.5	0.9	*Christensenellaceae*	2.9	1.6
Cutaneous mucus	*Burkholderiaceae*	16	1.6	*Burkholderiaceae*	14	1.3
	Alteromonadaceae	15	1.4	*Alteromonadaceae*	13	1.5
	Nitrincolaceae	13	1.2	*Lactobacillaceae*	12	2.6
	Lactobacillaceae	6.4	2.1	*Nitrincolaceae*	11	1.3
	Lachnospiraceae	5.8	1.6	*Lachnospiraceae*	9.0	1.7
	Beijerinckiaceae	5.4	0.6	*Beijerinckiaceae*	8.1	2.1
	Christensenellaceae	3.0	1.1	*Christensenellaceae*	5.7	1.2
	Rhodobacteraceae	2.9	0.4	*Ruminococcaceae*	2.6	0.5
	Clostridiaceae_1	2.5	1.9	*Rhodobacteraceae*	2.3	0.3
	Micrococcaceae	2.4	2.2	*Clostridiaceae_1*	1.7	1.0
Water	*Burkholderiaceae*	27	1.4			
	Sporichthyaceae	20	2.1			
	Chitinophagaceae	6.7	1.0			
	Beijerinckiaceae	5.1	0.5			
	Saccharimonadaceae	4.4	3.5			
	Methylophilaceae	3.8	0.4			
	Ferrovaceae	3.2	0.3			
	Pirellulaceae	2.9	0.4			
	Micropepsaceae	2.7	0.3			
	Mycobacteriaceae	2.2	1.4			

Tab. S7. Mean relative abundance (%) of the 25 most abundant prey found in the stomach content of stocked and wild parrs. S.e.m. Standard error of the mean.

**Wild**			**Stocked**		
**Species**	**mean (%)**	**s.e.m**	**Species**	**mean (%)**	**s.e.m**
Eurylophella versimilis	13.44	8.54	*Ephemerella invaria*	12.81	7.45
Hydropsyche versimilis	11.28	8.04	*Tipula sp.*	11.01	10.98
Promoresia versimilis	8.99	8.99	*Chironomidae sp.*	9.91	6.80
Multiple hits: Simulium truncatum, Simulium versimilis	8.60	8.60	*Lepidostoma pictile*	8.57	7.04
Tipula versimilis	8.38	8.35	*Epinotia sp.*	8.05	8.04
Baetis versimilis	7.24	4.53	*Protoboarmia porcelaria*	7.93	7.92
Amphigerontia versimilis	5.50	5.50	*Isoperla bilineata*	6.00	4.04
Brachycentrus versimilis	4.41	4.41	*Hydropsyche sparna*	5.56	5.54
Rhithrogena versimilis	3.76	2.96	*Leptophlebia sp.*	4.78	4.57
Multiple hits: Empididae sp., Neoplasta scapularis, Empididae versimilis	3.40	3.40	*Multiple hits: Megaselia sp., Phoridae sp.*	3.15	3.15
Bibio versimilis	3.06	3.06	*Optioservus sp.*	2.97	2.97
Neoplasta versimilis	3.05	3.05	*Eurylophella verisimilis*	2.44	1.99
Ephemerella versimilis	2.73	1.15	*Clastoptera obtusa*	2.13	2.10
Paracapnia versimilis	2.67	1.69	*Olophrum consimile*	2.07	2.05
Thienemanniella versimilis	2.43	2.34	*Maccaffertium vicarium*	1.91	1.89
Multiple hits: Empididae sp, Empididae versimilis	1.41	1.41	*Epinotia rectiplicana*	1.76	1.76
Heptagenia versimilis	1.39	1.27	*Thienemanniella xena*	1.07	0.84
Multiple hits: Hydropsyche aenigma, Hydropsyche versimilis	1.09	1.09	*Acerpenna pygmaea*	1.06	0.65
Gelis versimilis	0.90	0.90	*Hydroptila nr.*	1.01	0.69
Attenella versimilis	0.65	0.46	*Phoridae sp.*	0.74	0.74
Hydroptila versimilis	0.65	0.37	*Agapetus pinatus*	0.63	0.63
Acerpenna versimilis	0.64	0.49	*Pasiphaea multidentata*	0.44	0.44
Maccaffertium versimilis	0.56	0.52	*Isoperla cotta*	0.42	0.40
Leucrocuta versimilis	0.54	0.53	*Hydropsyche slossonae*	0.40	0.40
Chironomidae versimilis	0.52	0.49	*Platypeza sp.*	0.39	0.39

Tab. S8. Primer sequence for genetic assignation.

Informations sur les loci utilisés pour la caractérisation génétique. L'astérisque (*) indique l'amorce à laquelle est fixée la molécule fluorescente

Locis	Primer sequence (5'-3')	Binding temperature (°C)	Reference
Ssa85	F : AGGTGGGTCCTCCAAGCTAC<br>R : ACCCGCTCCTCACTTAATC*	58	(Oreilly et al., 1996)
Ssa171	F : TTATTATCCAAAGGGGTCAAAA<br>R : GAGGTCGCTGGGGTTTACTAT*	58	(Oreilly et al., 1996)
Ssa197	F : GGGTTGAGTAGGGAGGCTTG<br>R : TGGCAGGGATTTGACATAAC*	58	(Oreilly et al., 1996)
Ssa202	F : CTTGGAATATCTAGAATATGGC<br>R : TTCATGTGTTAATGTTGCGTG*	58	(Oreilly et al., 1996)
SsaD58	F : TAGAGTTTGTTCTCTGGCTTTG*<br>R : AGACCCTAGGACTGGCTACTG	58	(King et al., 2005)
SsaD71	F : AACGTGAAACATAAATCGATGG*<br>R : TAAGAATGGGTTGCCTATGAG	58	(King et al., 2005)
SsaD85	F : CTTTGGCTGTTTCAGGTATGAC*<br>R : CACTGCTCTACAACAGAAGTCTC	58	(King, comm. pers.)
SsaD144	F : TTGTGAAGGGGCTGACTAAC*<br>R : TCAATTGTTGGGTGCACATAG	58	(King et al., 2005)
SsaD486	F : TCGCTGTGTATCAGTATTTTGG*<br>R : ACTCGGATAACACTCACAGGTC	58	(King et al., 2005)
Ssosl417	F : TTGTTCAGTGTATATGTGTCCCAT*<br>R : GATCTTCACTGCCACCTTATGACC	58	(Slettan et al., 1995)
SsspG7	F : CTTGGTCCCGTTCTTACGACAACC*<br>R : TGCACGCTGCTTGGTCCTTG	58	(Paterson et al., 2004)
Sssp1605	F : CGCAATGGAAGTCAGTGGACTGG*<br>R :<br>CTGATTTAGCTTTTTAGTGCCCAATGC	58	(Paterson et al., 2004)
Sssp2201	F : TTTAGATGGTGGGATACTGGGAGGC*<br>R :<br>CGGGAGCCCCATAACCCTACTAATAAC	58	(Paterson et al., 2004)
Sssp2210	F :<br>AAGTATTCATGCACACACATTCACTGC*<br>R : CAAGACCCTTTTTCCAATGGGATTC	58	(Paterson et al., 2004)
Sssp2215	F : ACTAGCCAGGTGTCCTGCCGGTC*<br>R : AGGGTCAGTCAGTCACACCATGCAC	58	(Paterson et al., 2004)
Sssp2216	F :<br>GGCCCAGACAGATAAACAAACACGC*<br>R : GCCAACAGCAGCATCTACACCCAG	58	(Paterson et al., 2004)
MST-3	F : CCCTGGTTTGACTTTGTCTCA*<br>R : AGGCACTCTCACCAGCTAAAGATG	58	(Presa et Guyomard, 1996)

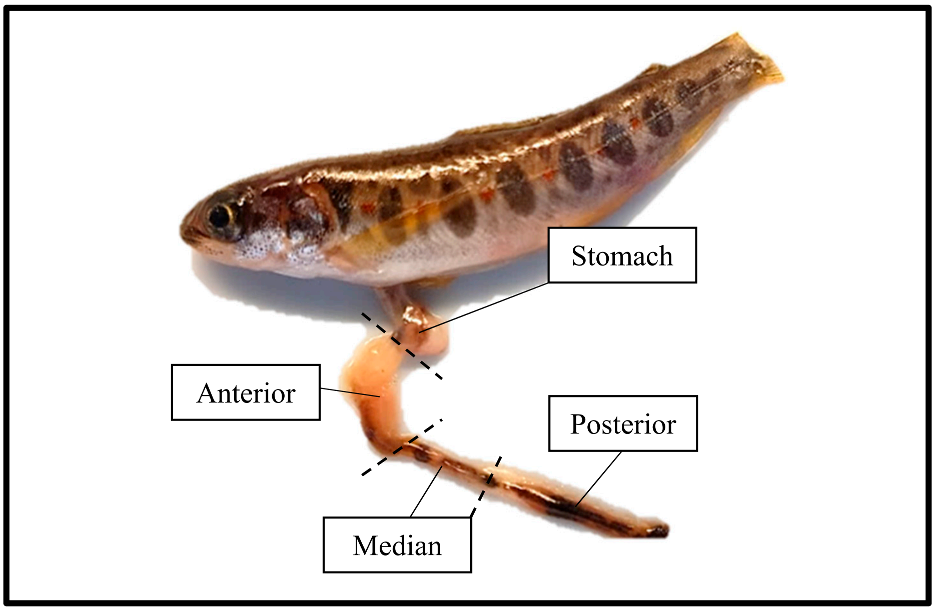

Fig S1. Body compartments and gut segments that were separated for the microbiota analysis of the intestinal tract.

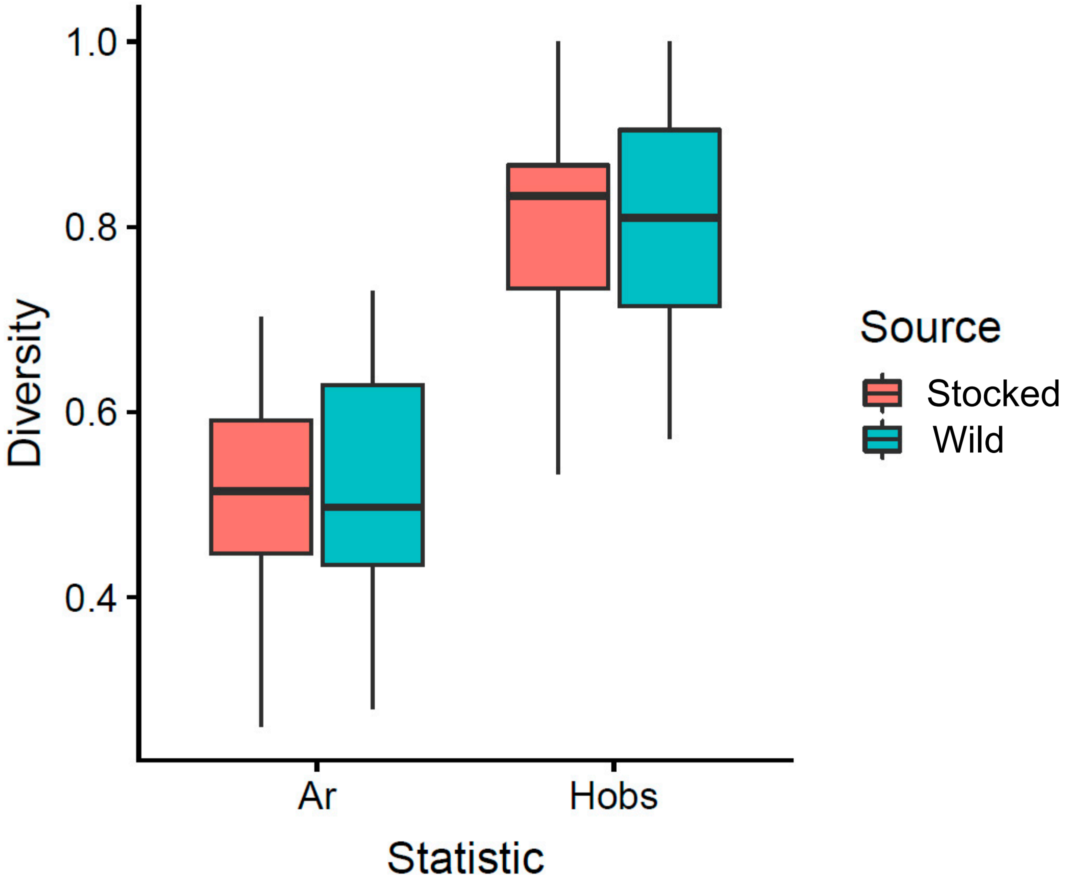

Fig S2. Genetic diversity for stocked and wild parrs calculated with individuals from year 2016 and 2017. Ar ; Allelic richness, Hobs ; Observed heterozygosity.

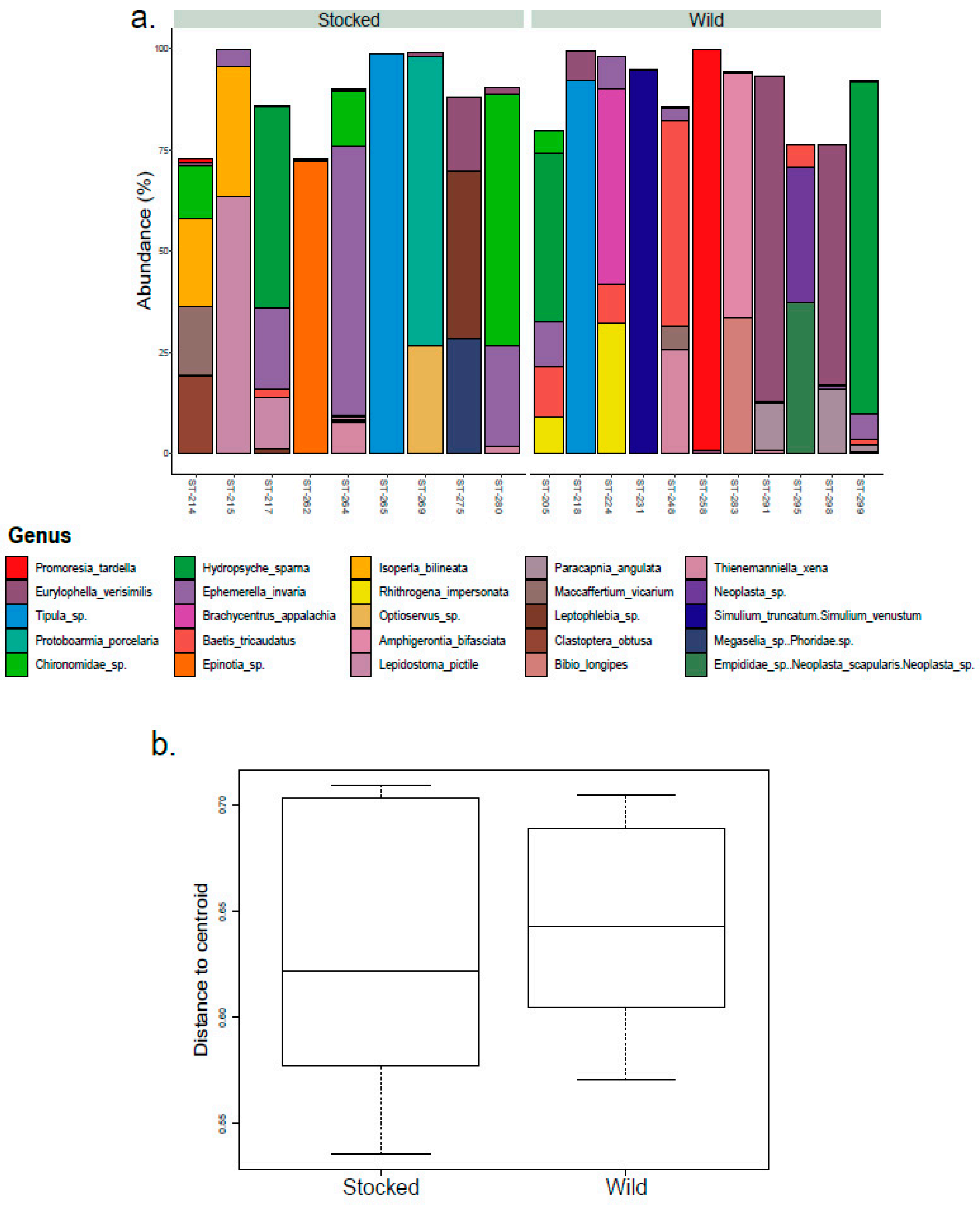

Fig. S3. The 25 most abundant eukaryotic species identified by the amplification of the biomarker Cytochrome Oxydase I (COI) from stocked and wild parrs' stomach contents (**a**) highlight a high interindividual variability between both groups (**b**). The boxplot represents the distance to the centroid of the Bray Curtis distances whithin each group, based on an analysis of the multivariate homogeneity of dispersion (variances) of samples.

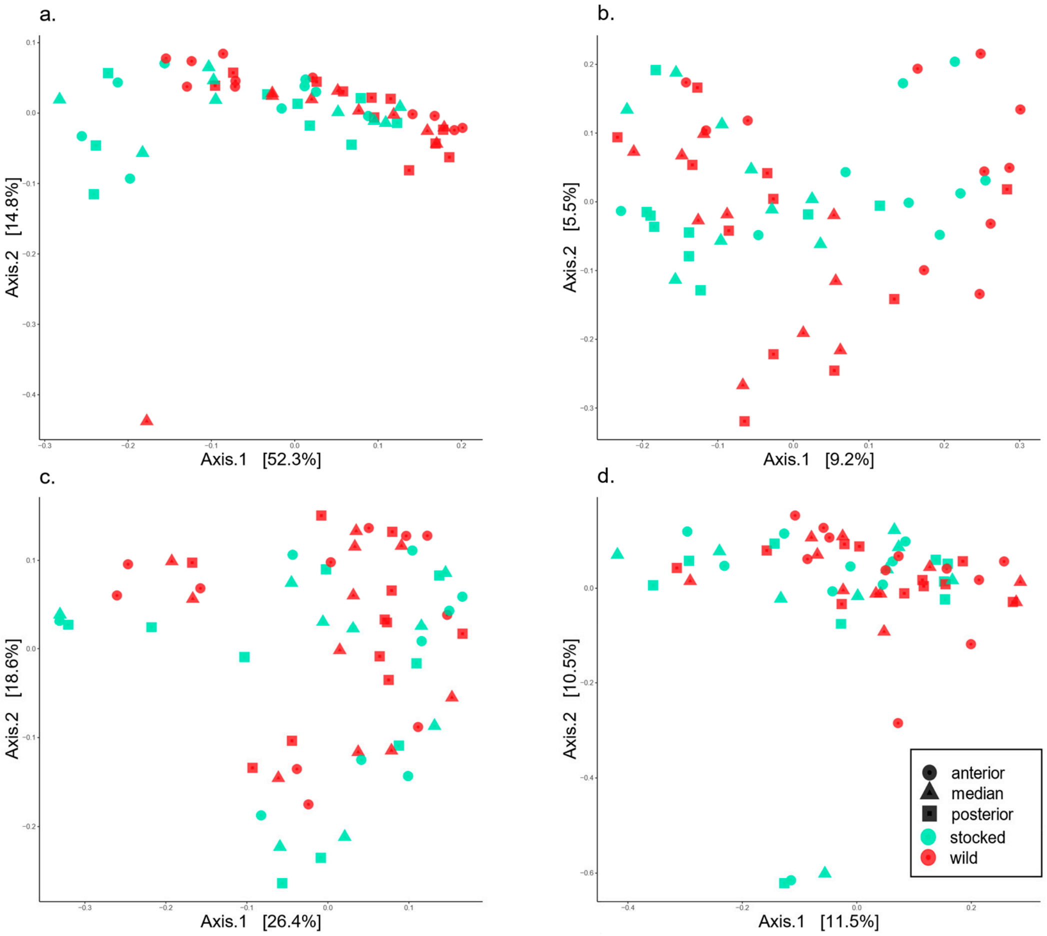

Fig. S4. Principal Coordinates Analysis (PCoA) highlights distinct patterns of microbial community structure of gut and digesta samples for weighted (a, c) or unweighted (b, d) UniFrac distances. **a.** and **b.** show PCoA from weighted (**a**) and unweighted (**b**) Unifrac distances from gut samples, where bacterial community composition calculated with weighted UniFrac (a) is significantly different between stocked and wild parrs (F = 5.8617, *p* = 0.003), indicating that abundance of shared bacteria significantly differs. **c.** and **d.** show PCoA from weighted (**c**) and unweighted (**d**) UniFrac distances from digesta samples, where bacterial community composition calculated with unweighted UniFrac (d) is significantly different between stocked and wild parrs (F = 1.8704, *p* = 0.002), indicating that digesta community differ by the presence/absence of bacterial strains.
